# A proof of concept study of Infliximab for the treatment of HTLV-1-associated myelopathy

**DOI:** 10.1186/1742-4690-11-S1-P31

**Published:** 2014-01-07

**Authors:** Fabiola Martin, Hannah Castro, Carolyn Gabriel, Adine Adonis, Alexandra Fedina, Linda Harrison, Liz Brodnicki, Maria A Demontis, Abdel G Babiker, Jonathan N Weber, Charles RM Bangham, Graham P Taylor

**Affiliations:** 1 Centre for Immunology and Infection, Department of Biology, Hull and York Medical School, University of York, York, UK; 2 Medical Research Council, Clinical Trials Unit, London, UK; 3 Department of Neurology, St Mary’s Hospital, London, UK; 4 National Centre for Human Retrovirology, St Mary’s Hospital, London, UK; 5 Section of Infectious Diseases, Faculty of Medicine, Imperial College, UK

## Background

Disease modifying treatment options for patients with HAM are limited. Most studies have included all patients regardless of duration, disability or disease activity. The Medical Research Council UK funded a series of proof of concept studies for patients with early (50% deterioration during preceding 3 months). The results of the first study of ciclosporin have been published. The second study, of the anti-TNF monoclonal antibody Infliximab, is presented.

## Study design

Open-label, clinical endpoint study of Infliximab 3mg/kg infused intravenously at weeks 0, 2 and 8 and then every 8 weeks until and including week 40 of the study. Primary endpoints were time to and incidence of clinical failure.

## Results

The study was terminated early for futility. Three patients were recruited to and completed the study. Patient #1 developed a rash and headache after the 2nd infusion. This was considered to be immune complex mediated. Patient #2 experienced hypersensitivity reaction with profound hypotension during the 4th infusion (week 16). In both cases treatment was discontinued. Patient #3 completed all 7 infusions without adverse effect. Reduced spasticity was observed in all 3 patients during on-treatment period. Patient #1 was unable to stand throughout the study. 10m timed walk improved in Patient #2 but deteriorated in #3. No trend was observed in pain scores, β2M or HTLV-1 viral load in blood and CSF.

## Conclusion

High severe adverse event rate with Infliximab therapy was observed. Future studies should co-administer with methotrexate as per the treatment of rheumatoid arthritis.

